# Combinatorial inhibition of BTK, PI3K-AKT and BRD4-MYC as a strategy for treatment of mantle cell lymphoma

**DOI:** 10.1186/s43556-021-00066-9

**Published:** 2022-01-15

**Authors:** Kendra R. Vann, Dhananjaya Pal, Audrey L. Smith, Namood-e Sahar, Maddeboina Krishnaiah, Dalia El-Gamal, Tatiana G. Kutateladze

**Affiliations:** 1 Department of Pharmacology, University of Colorado School of Medicine, Aurora, CO USA; 2 Molecular Targeted Therapeutics Laboratory, Levine Cancer Institute, Charlotte, NC USA; 3 Division of Hematology and Oncology, Department of Pediatrics, Moores Cancer Center, University of California San Diego, La Jolla, CA USA; 4 Eppley Institute for Research in Cancer and Allied Diseases, Fred & Pamela Buffett Cancer Center, University of Nebraska Medical Center, Omaha, NE USA

**Keywords:** Mantle cell lymphoma, BRD4, Bromodomain, Inhibitor, Bruton's tyrosine kinase, Phosphatidylinositol-3 kinase

## Abstract

Mantle cell lymphoma (MCL) is a subtype of non-Hodgkin’s lymphoma characterized by poor prognosis. The complexity of MCL pathogenesis arises from aberrant activities of diverse signaling pathways, including BTK, PI3K–AKT–mTOR and MYC-BRD4. Here, we report that MCL-related signaling pathways can be altered by a single small molecule inhibitor, SRX3305. Binding and kinase activities along with resonance changes in NMR experiments reveal that SRX3305 targets both bromodomains of BRD4 and is highly potent in inhibition of the PI3K isoforms α, γ and δ, as well as BTK and the drug-resistant BTK mutant. Preclinical investigations herein reveal that SRX3305 perturbs the cell cycle, promotes apoptosis in MCL cell lines and shows dose dependent anti-proliferative activity in both MCL and drug-resistant MCL cells. Our findings underscore the effectiveness of novel multi-action small molecule inhibitors for potential treatment of MCL.

**Supplementary Information:**

The online version contains supplementary material available at 10.1186/s43556-021-00066-9.

## Introduction

Mantle cell lymphoma (MCL) is a mature B cell malignancy with characteristic t(11;14)(q13;q32) translocation juxtaposing *IGH* and *CCND1* gene loci [1]. This translocation leads to overexpression of cyclin D1 and dysregulation of the cell cycle at the G1/S phase transition. While treatment with combination chemotherapeutic regimens is initially effective, nearly all patients relapse with a median survival of only 2.5 – 5 years [2, 3]. Several therapeutic options have been pursued to improve the outcome for MCL, including combination chemotherapy, high-dose chemotherapy followed by autologous stem cell transplant (ASCT) and monoclonal antibody therapy, all of which have been met with minimal success. Outside of ASCT, a therapy for which most patients are ineligible, MCL remains incurable to date. In recent years, several small molecule inhibitors have emerged for B-cell malignancies to provide improved survival outcomes with diminished therapy-associated morbidities. The therapeutic options for MCL are constantly growing in the era of novel targeted therapies – Ibrutinib, Acalabrutinib and Venetoclax – and a shift to chemotherapy-free regimens is highly warranted [3].

Aberrant B-cell receptor (BCR) signaling is a key mediator of MCL disease pathogenesis and contributes to enhanced survival and proliferation of malignant B-cells [4, 5]. Agents targeting Bruton's tyrosine kinase (BTK), a core effector of the BCR signaling pathway (e.g., Ibrutinib) [6–8] and other BCR pathway members, such as phosphatidylinositol-3 kinase δ (PI3K-δ; e.g., Idelalisib) [9], have demonstrated high response rates in MCL [10]; however, these responses were not durable, suggesting a combinatory approach to enhance patient outcome. Despite the high overall response rate seen in relapsed/refractory (R/R) MCL patients treated with Ibrutinib (68%), only 21% of patients reached a complete response with a median progression free survival of ~14 months [6]. Resistance to Ibrutinib is common, and there are few approved therapies capable of improving MCL patient outcome to an extent, making the post-FDA approved R/R MCL treatment setting an unmet clinical need [11].

Dysregulation of MYC plays an essential role in the pathogenesis of B-cell lymphomas, including MCL, and is associated with poor prognosis and aggressive clinical behavior [12–14]. Pharmacological inhibition of the BET family proteins BRD2, BRD3, BRD4, and BRDt has emerged as a therapeutic approach to target MYC-dependent transcription [15, 16] because BRD4 regulates *MYC* and a number of other lymphoma-relevant oncogenes (BCL2, CDK4/6, and cyclin D1) [17–21]. BRD4 is also essential for the transcriptional activity of NF-κB downstream of BCR signaling [22, 23], which makes it a vital therapeutic target in MCL. Various small molecule BET inhibitors, such as JQ1, I-BET-151 and OTX-015, have demonstrated anti-tumor activity in preclinical models of B-cell malignancies [24, 25], including MCL [20, 26]. Notably, due to the reported synergy with Ibrutinib, BET inhibitors may serve as a therapeutic option for MCL in the setting of Ibrutinib resistance [20, 26]. Recent studies have demonstrated the effectiveness of targeting multiple disease-relevant pathways with a combination of inhibitors (PI3Ki and BETi/BTKi) or with a single multi-action agent (PI3K-BET and PI3K-BRD4-CDK4/6). These therapeutic approaches are becoming an attractive way to achieve a durable response and combat drug resistance [10, 21, 27–29]. We recently introduced thienopyranone scaffold-based chemotypes for the combinatorial inhibition of BTK, PI3K-AKT and BRD4-MYC [30]. Here, we report on SRX3305, a second generation of the highly potent BTK/PI3K/BRD4 triple action inhibitors and demonstrate the effectiveness of this approach for potential treatment of MCL.

## Results

### SRX3305 binds to BRD4 and inhibits BTK and PI3K

**Fig. 1:**
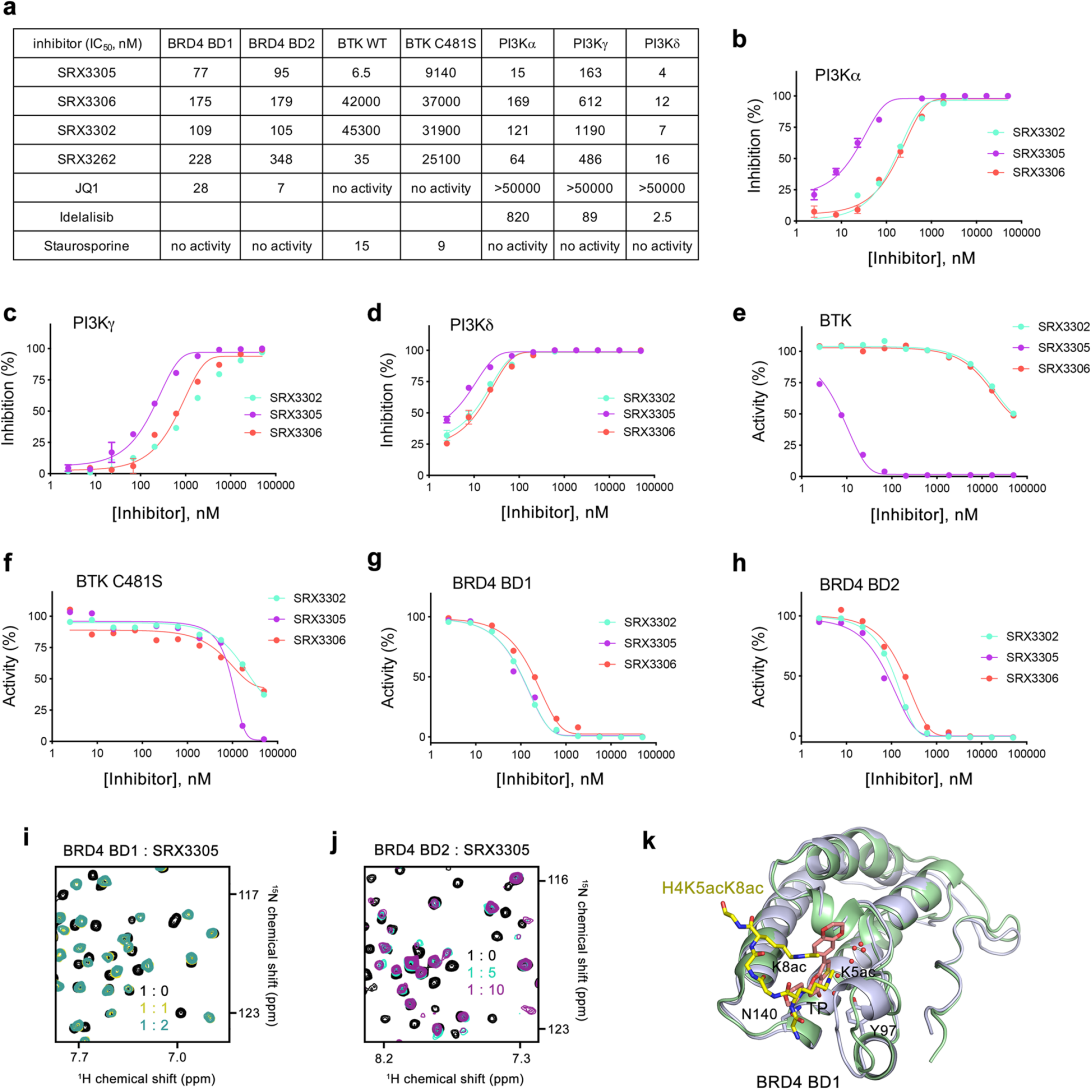
SRX3305 is a potent triple activity BTK/PI3K/BRD4 inhibitor. **a** IC_50_ values (nM) of SRX3305 and other indicated inhibitors of BRD4, PI3K and BTK as measured by displacement binding and kinase assays. Values for SRX3262 are from [30] and values for Idelalisib are from [32]. **b-h** IC_50_ curves for indicated targets by indicated inhibitors. **i, j** Superimposed ^1^H,^15^N HSQC spectra of uniformly ^15^N-labeled BRD4 BD1 and BRD4 BD2, recorded while SRX3305 was titrated in. The spectra are color-coded according to the protein: inhibitor molar ratio (inset). **k** Structural overlay of the complexes: BRD4 BD1 (light blue) with the TP inhibitor SF2523 (pink) (PDB: 5U28) [27] and BRD4 BD1 (light green) with H4K5acK8ac peptide (yellow) (PDB: 3UVW) [33]. Water molecules are shown as red spheres

In effort to develop small molecule agents which act against MCL, we designed a series of thienopyranone (TP)-based compounds with the ability to concomitantly bind bromodomains (BDs) of BRD4, the catalytic domain of BTK, and the catalytic domain of PI3K. We have demonstrated that dual and triple action TP-scaffold compounds show promising results as anti-cancer agents [27–29], and the first generation of BTK/PI3K/BRD4 inhibitors, such as SRX3262, was cytotoxic to MCL cells [30]. To enhance efficacy of these compounds, a new set of chemotypes was generated [31] and screened in Alpha Screen and kinase assays. Among the new set of inhibitors, SRX3305 showed a substantial increase in potency for all three targets, BTK, PI3K and BRD4 compared to SRX3262 (Fig. 1a). SRX3305 displayed an IC_50_ of 6.5 nM, 15 nM, and 4 nM toward BTK, PI3Kɑ and PI3Kδ, respectively, and was a ~4-5-fold more potent inhibitor than SRX3262 towards these kinases (Fig. 1a-e). SRX3305 also retained its high inhibitory activity toward both bromodomains of BRD4, bromodomain 1 (BD1) and bromodomain 2 (BD2) and was ~2-fold more potent than SRX3262 (Fig. 1a, g, h). Importantly, SRX3305 showed enhanced binding to the drug-resistant C481S mutant of BTK compared to SRX3262 (IC_50_=9 μM and 25 μM, respectively), was more effective than the pan-kinase inhibitor Staurosporine for WT BTK (IC_50_=15 nM) and elicited an effect comparable to the PI3K inhibitor Idelalisib (IC_50_=2.5 nM) [32] on PI3Kδ (Fig. 1a, f).

To gain insight into the mechanism of inhibition of BRD4, we produced ^15^N-labeled BRD4 BD1 and BRD4 BD2 and examined the binding of SRX3305 to each domain by NMR spectroscopy. Gradual addition of SRX3305 to either the BRD4 BD1 NMR sample or BRD4 BD2 NMR sample induced substantial chemical shift changes in ^1^H,^15^N heteronuclear single quantum coherence (HSQC) spectra of BD1 and BD2, indicating that both domains are targets of SRX3305 (Fig. 1i, j). These resonance changes were in slow exchange regime on the NMR timescale and suggested tight binding of SRX3305, confirming the nanomolar IC_50_ values, 77 nM for BD1 and 95 nM for BD2. Moreover, the pattern of these changes was similar to the pattern of changes observed in ^1^H,^15^N HSQC spectra of these domains due to the binding of other TP-scaffold compounds [27–29], including SF2523, as well as acetylated peptides [33], implying that bound SRX3305 occupies the acetyllysine-binding sites of BRD4 BD1 and BRD4 BD2 (Fig. 1k).

### SRX3305 is cytotoxic to MCL cells

**Fig. 2:**
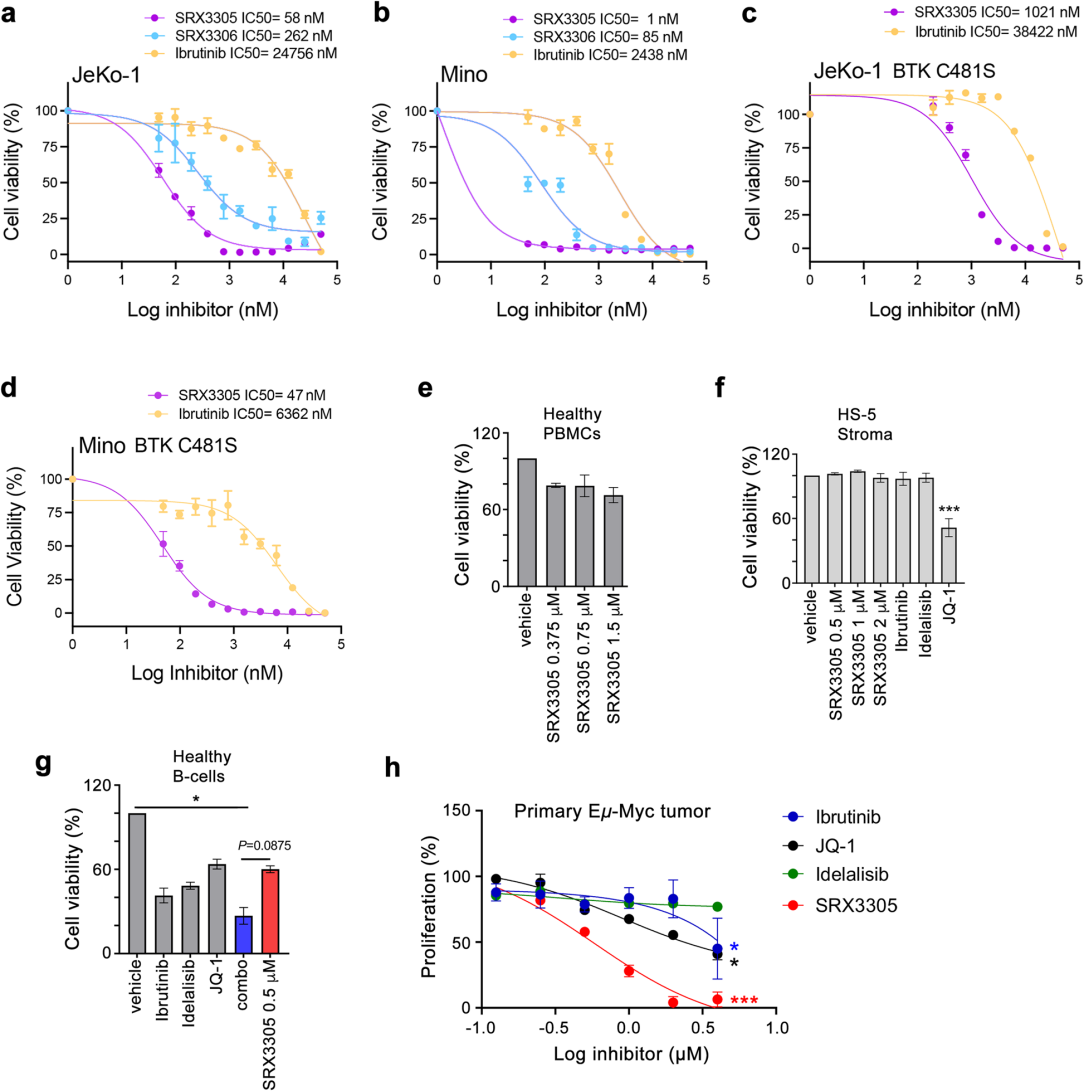
SRX3305 is cytotoxic to MCL cells. **a-d** Anti-proliferative activity of JeKo-1, Mino, JeKo-1 BTK C481S and Mino BTK C481S MCL cell lines treated with increasing concentrations of indicated inhibitors. Error bars represent mean ± SEM (*n*= independent 3 experiments). **e** PBMCs from healthy subjects (*n* = 4) were treated with increasing concentrations of SRX3305 or DMSO vehicle for 48 h. Viability was assessed via MTS assay and results are normalized to vehicle. **f** Human HS-5 bone marrow-derived stromal cell line was treated for 72 h with DMSO vehicle, indicated concentrations of SRX3305, 1 μM Ibrutinib, 1 μM Idelalisib or 1 μM JQ-1. Viability was assessed via MTS assay and results are normalized to vehicle. Results (mean ± SEM) are given from *n* = 4 independent experiments. *** *P*< 0.001 *vs.* vehicle. **g** B-cells were purified from healthy subject PBMCs (*n* = 3) using negative selection kit. Normal B-cells were then incubated with DMSO vehicle, 0.5 μM Ibrutinib, 0.5 μM Idelalisib, 0.5 μM JQ-1, a combination (combo) of the single inhibitors (Ibrutinib, Idelalisib, and JQ1 each at 0.5 μM), or 0.5 μM SRX3305. After 48 h, viability was assessed by MTS. Results (mean ± SEM) were normalized to vehicle. * *P*< 0.05 *vs.* vehicle and as indicted for combo *vs.* SRX3305. **h** Splenocytes isolated from morbid E*μ*-Myc mice (*n* = 4) were stimulated *ex vivo* with PMA/Ionomycin and treated with DMSO vehicle or increasing inhibitor concentrations as indicated. After 48 h, proliferation was assessed by MTS and normalized to vehicle. Results are shown as (mean ± SD). Dose-dependent decrease in cell proliferation is observed with symbols denoting significance (* *P*< 0.05, *** *P*< 0.001)

To establish the anti-cancer activity of SRX3305, we performed cytotoxicity experiments treating the MCL cell lines JeKo-1, Mino, Granta, JeKo-1 BTK C481S and Mino BTK C481S with increasing concentrations of SRX3305, SRX3306 or the BTK inhibitor Ibrutinib. The dose response curves generated from alamarBlue and CellTiter-Glo cell viability assays revealed strong anti-proliferative function of SRX3305 (Fig. 2a-d and Supplementary Fig. 1). SRX3305 showed an IC_50_ of 1 nM in Mino cells, 58 nM in JeKo-1 cells and 1.1 μM in Granta cells and had ~5-80-fold higher efficacy in these cell lines compared to SRX3306 and SRX3262. Moreover, SRX3305 was substantially more cytotoxic than Ibrutinib to these MCL cells due to the concurrent inhibition of not only BTK but also two additional targets, PI3K and BRD4. Cell viability assays using the JeKo-1 BTK C481S and Mino BTK C481S mutant cell lines further demonstrated that SRX3305 has higher efficacy (IC_50_=1 μM and 47 nM, respectively) compared to Ibrutinib (IC_50_=38 μM and 6 μM, respectively) (Fig. 2c, d). SRX3305 was minimally toxic to healthy donor peripheral blood mononuclear cells (PBMCs) (Fig. 2e) or bystander healthy stromal cells (Fig. 2f) and was less toxic to healthy donor B-cells when compared to the combination of single-target drugs (combo) required to effectively inhibit the same three targets (Fig. 2g). Furthermore, the anti-tumor effect of SRX3305 was confirmed in primary E*μ*-Myc tumor samples (Fig. 2h).

### SRX3305 shows improved efficacy in MCL and Ibrutinib-resistant MCL cells

**Fig. 3:**
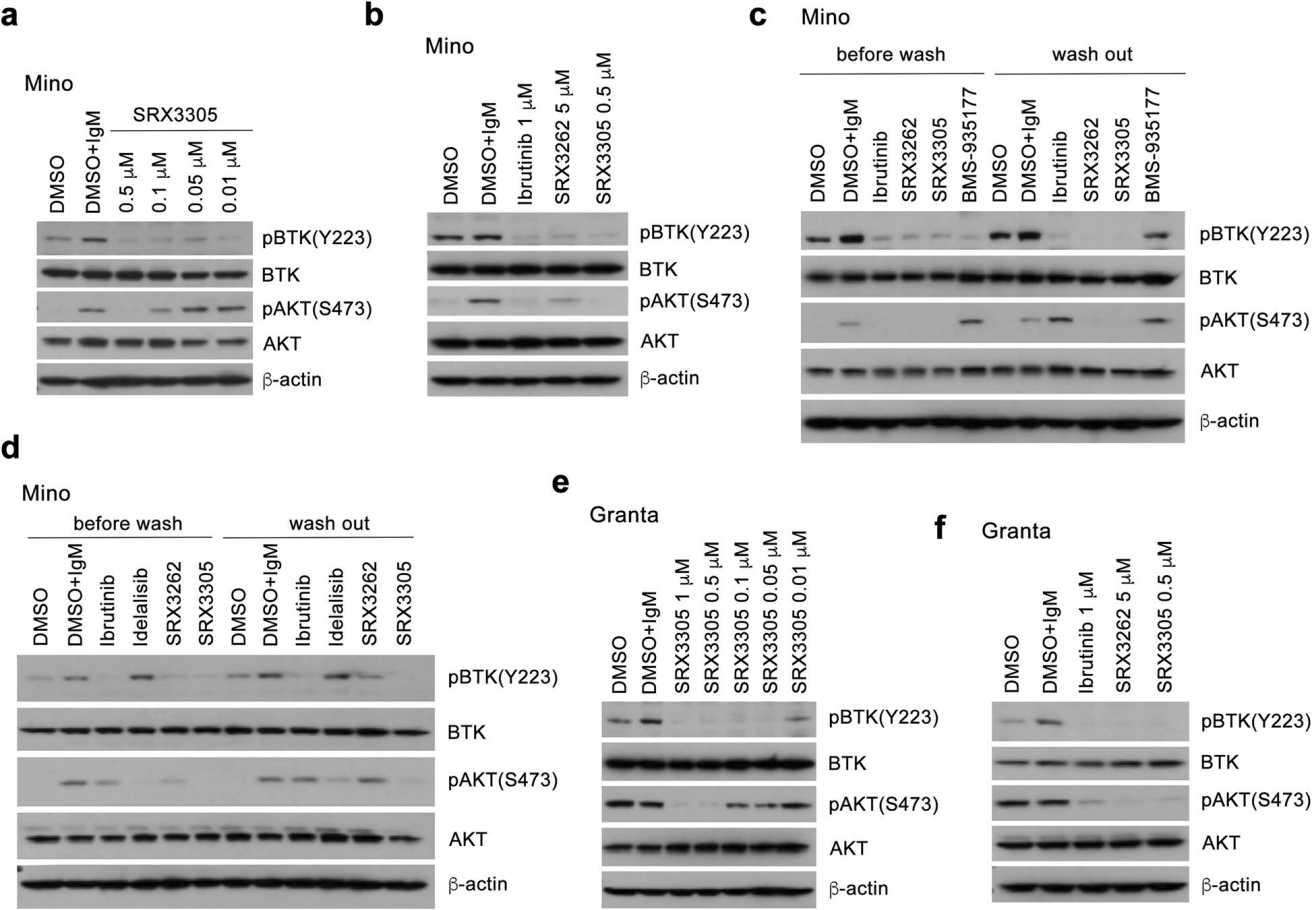
SRX3305 is efficacious in MCL and Ibrutinib-resistant MCL cells. **a**, **b** Western blot analysis of lysates from IgM-stimulated Mino cells treated with the indicated concentrations of Ibrutinib, SRX3305 or SRX3262 for 1 h. BTK and PI3K signaling was assessed by the levels of BTK, phosphorylated at Y223 BTK [pBTK(Y223)], AKT, and phosphorylated at S473 AKT [pAKT(S473)]. **c**, **d** Western blots analysis of lysates from IgM-stimulated Mino cells without and with washing out indicated inhibitors 24 h post treatment. **e**, **f** Western blot analysis of lysates from IgM-stimulated Granta cells treated with increasing concentrations of SRX3305, SRX3262 or Ibrutinib for 1 h

The effect of SRX3305 on activation of BTK/PI3K signaling was examined in IgM stimulated JeKo-1 and Mino MCL cells using western blot analysis. Treatment of the cells with SRX3305 blocked activation of both BTK and AKT (a downstream effector of PI3K) as evidenced by the decrease of BTK phosphorylation at Tyr223 and AKT phosphorylation at Ser473 (Fig. 3a and Supplementary Fig. 2a). SRX3305 at a concentration of 0.5 μM inactivated the BTK/PI3K signaling to the same extent as the BTK inhibitor Ibrutinib at a concentration of 1 μM and SRX3262 at a concentration of 5 μM (Fig. 3b).

We next tested whether SRX3305 covalently binds to BTK. We treated Mino cells with SRX3305, SRX3262, Ibrutinib (irreversible covalent BTK inhibitor) and BMS-935177 (reversible BTK inhibitor) for 1 h and then performed inhibitor wash out experiments (Fig. 3c, d). As expected, cells treated with the reversible inhibitor BMS-935177 regained BTK phosphorylation after extensive washes with PBS buffer; however, cells treated with SRX3305, SRX3262 and Ibrutinib retained inhibition of BTK phosphorylation. SRX3305 and SRX3262 also retained inhibition of AKT phosphorylation in contrast to Ibrutinib in Mino cells (Fig. 3c) but not in the Mino BTK C481S mutant cells (Supplementary Fig. 2b, c). Furthermore, SRX3305 showed dose dependent anti-proliferative activity in Granta, an Ibrutinib-resistant cell line, and again it was as potent at a concentration of 0.5 μM as SRX3262 at a concentration of 5 μM (Fig. 3e, f). Collectively, these data demonstrate that SRX3305 is an irreversible inhibitor, which displays augmented efficacy in both MCL and Ibrutinib-resistant MCL cell lines.

### SRX3305 impacts gene expression, perturbs the cell cycle and promotes apoptosis in MCL

**Fig. 4:**
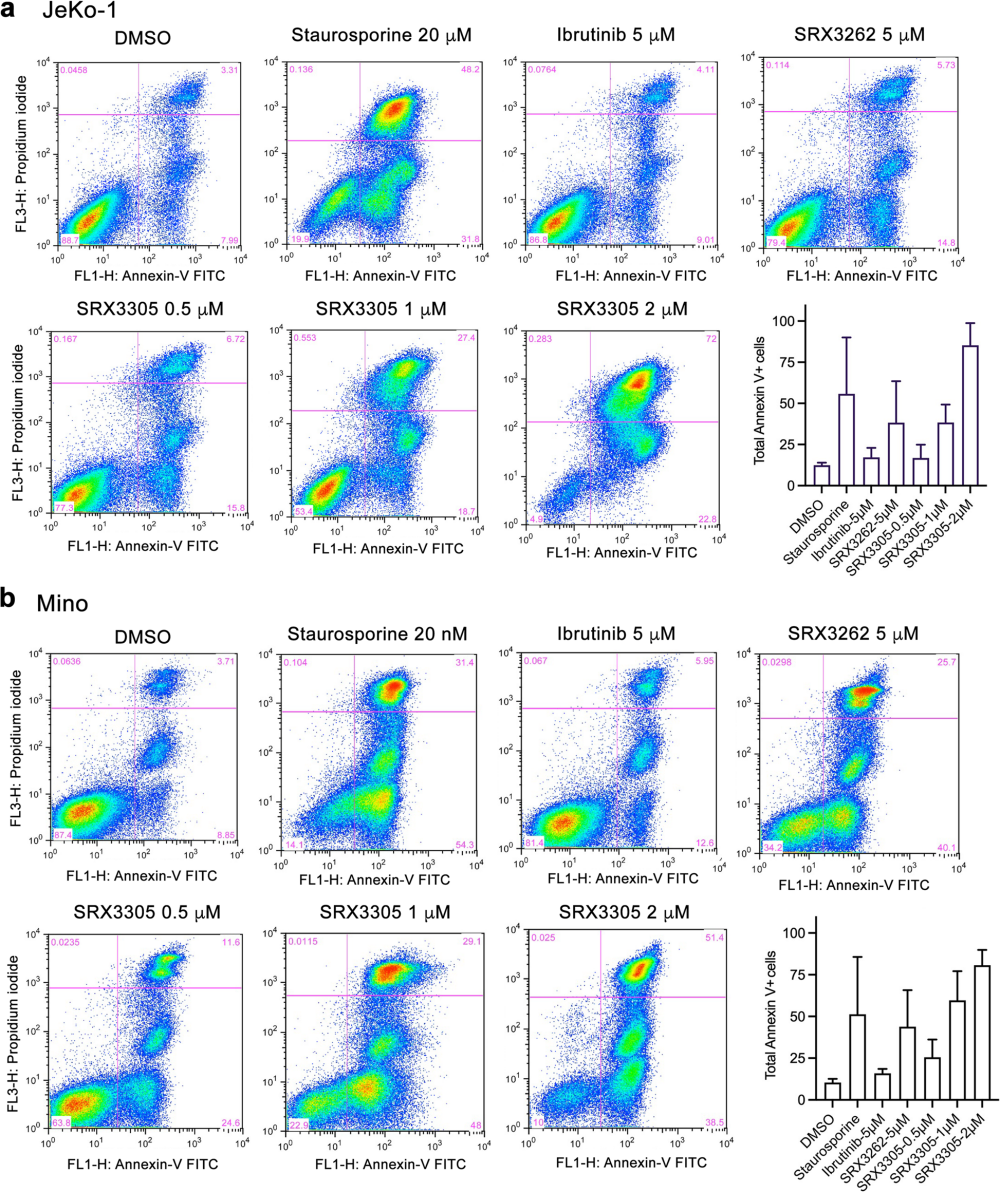
SRX3305 induces apoptosis and cell cycle arrest. **a**, **b** Flow cytometry analysis of apoptosis induced in the JeKo-1 (**a**) and Mino (**b**) MCL cell lines treated with control (DMSO) or the inhibitors Staurosporine, Ibrutinib, SRX3262 and SRX3305 and assessed after 24 h by evaluating the total percentage of Annexin V/ Propidium Iodide positively stained cells. Histograms show % apoptotic cells (Annexin V positive cells). Experiments were independently performed in duplicate (see also Supplementary Figs. 4 and 5)

BRD4 is known to regulate expression of target genes, including *MYC* and other oncogenes [16, 34]. We treated JeKo-1 cells with SRX3305, the BRD4 inhibitor PLX51107, or control DMSO and analyzed expression of *cMYC* and *HEXIM1*, a pharmacodynamic marker for BRD4 inhibition, by qRT-PCR. As expected, treatment with SRX3305 led to a decrease in expression of *cMYC* and an increase in expression of *HEXIM1* (Supplementary Fig. 3). Given the synergistic relationship of BRD4, PI3K and BTK, we assessed the apoptotic response induced by SRX3305. Treatment of JeKo-1 and Mino cells with increasing concentrations of SRX3305, up to 2 μM for 24 h, stimulated apoptosis, as measured by Annexin V and propidium incorporation in flow cytometry (Fig. 4 and Supplementary Figs. 4 and 5). SRX3305 at 2 μM induced apoptosis in JeKo-1 and Mino cells to the same extent as Staurosporine at 20 nM, whereas Ibrutinib showed no significant effect in JeKo-1 cells and a mild effect in Mino cells at a concentration of 5 μM. SRX3305 treatment of JeKo-1 cells led to alterations in the cell cycle phases with cell population in S phase increasing from 49 to 65% and cell population in G2 phase increasing from 8 to 18% (Supplementary Fig. 6). The effect was milder in Mino cells, where cell population in G2 phase increased from 12 to 19%. Together, these results indicate that SRX3262 inhibits MCL cell proliferation through inducing S/G2 phase cell cycle arrest and promoting apoptosis.

## Discussion

Given the absence of curative therapy for MCL, especially for patients with R/R disease, it is essential to explore new treatment options and strategies. Current anti-cancer treatments, such as the use of combinations of drugs to target multiple pathways, often suffer from additive toxicity, underscoring an urgent need for the development of potent anti-MCL agents with reduced drug-induced adverse effects. This could be achieved by developing highly specific inhibitors that concomitantly hit two or more major targets in MCL pathogenesis and thus provide a synergistically lethal effect.

In this study we have developed the most potent to date BRD4/PI3K/BTK inhibitor, SRX3305, that shows enhanced preclinical efficacy in MCL cells and overcomes Ibrutinib resistance by inducing cytotoxic mediated cell death in the acquired Ibrutinib resistance cells. SRX3305 is a single agent which simultaneously binds to the catalytic domains of BTK and PI3K and bromodomains of BRD4 and inhibits the critical signaling pathways known to be aberrantly activated in MCL, including BTK, PI3K–AKT–mTOR and MYC-BRD4.

Our findings demonstrate that SRX3305 perturbs the cell cycle, shows dose dependent anti-proliferative activity and promotes apoptosis in MCL cells. Moreover, *ex vivo* studies using primary E*μ*-Myc tumor samples confirm the anti-tumor activity of SRX3305 in an aggressive model of B-cell lymphoma. Further studies will focus on testing efficacy and toxicity of SRX3305 across various B-cell malignancies, including *in vivo* models, and on the development of the next generation of triple action anti-MCL agents.

## Materials and methods

### Cell lines, primary donor samples and reagents

Human Mantle Cell Lymphoma (MCL) cell lines Mino, JeKo-1 and Granta-519 (Granta) were received from Thomas Kipps and David Weinstock labs. BTK C481S mutant was cloned into a pMSCV-ires-tdTomato expression vector using the Gateway vector conversion system (Invitrogen). Cells were transfected and selected for homogeneity. Parental Mino and JeKo-1 cells served as WT BTK. Mino and JeKo-1 cells were grown in RPMI-1640 with 20% fetal bovine serum (FBS) and Granta cells were grown in Dulbecco’s modified Eagle’s medium (DMEM) containing 10% FBS. All cells were supplemented with 1% penicillin/streptomycin and incubated at 37 °C with 5% CO_2_. Cell lines were tested for mycoplasma and checked for authenticity against the ICLAC (International Cell Line Authentication Committee; http://iclac.org/databases/cross-contaminations/) list. Healthy donor peripheral blood mononuclear cells (PBMCs) were obtained from the Elutriation Core Facility per approved IRB protocol at the University of Nebraska Medical Center. For a subset of experiments, B-cells were purified from healthy donor PBMCs using B cell negative selection kit from StemCell Technologies (Vancouver, Canada) following manufacture’s protocol. Healthy donor cells were cultured in RPMI-1640 supplemented with 100 μg/mL streptomycin (P/S), 100 U/mL penicillin, 2 μM L-glutamine and 10% heat-inactivated fetal bovine serum (hi-FBS). Human HS-5 stromal cell line was obtained from the ATCC and cultured in DMEM supplemented with P/S and 10% hi-FBS. All antibodies were purchased from Cell Signaling Technology. Ibrutinib, Staurosporine, Idelalisib (CAL101) and JQ1 were purchased from Selleck Chemicals. SRX3305, SRX3306, SRX3302 and SRX3262 were synthesized and provided by SignalRx Pharmaceuticals, Inc. (Cumming, GA).

### Binding and kinase assays

Adapta kinase activity assays were carried out by ThermoFisher Scientific (Waltham, MA) to measure IC_50_ of the indicated inhibitors for PI3K isoforms. Kinase activity assays were carried out by Reaction Biology (Malvern, PA) to measure IC_50_ of the indicated inhibitors for BTK WT and BTK C481S (Ibrutinib-resistant mutant). Alpha Screen assays were carried out by Reaction Biology (Malvern, PA) to measure IC_50_ of the indicated inhibitors for His-tagged BRD4 BD1 and BD2 bromodomains.

### Protein expression and purification

The human pGEX6P-BRD4 BD1 (aa 43-180) and pGEX4T-BRD4 BD2 (aa 342-460) constructs were generated as previously described [28]. Briefly, BRD4 BD1 and BD2 bromodomain constructs were expressed in *E. coli* BL21 (DE3) RIL in M19 minimal media supplemented with ^15^NH_4_Cl and purified as GST fusions. Cells were induced with IPTG at a final concentration of 1 mM and grown overnight at 16 °C. The cells were harvested by centrifugation, resuspended in 10 mM HEPES pH 7.5, 150 mM NaCl, 1 mM TCEP, and lysed by sonication. The proteins were purified using glutathione Sepharose 4B beads, and the GST tag was cleaved with PreScission protease. The proteins were purified using a S100 column (GE Healthcare), equilibrated in 10 mM HEPES pH 7.5, 1 mM TCEP and 150 mM NaCl. The fractions of BRD4 BD1 and BD2 were assessed for purity by SDS-PAGE, buffer exchanged into PBS pH 6.8 and concentrated to ~10-20 mg/mL.

### NMR spectroscopy

NMR spectroscopy experiments were performed on a Varian INOVA 600 MHz spectrometer. Experiments were carried out on uniformly ^15^N-labeled BRD4 BD1 (0.15 mM) and BRD4 BD2 (0.25 mM) in PBS buffer pH 6.8, containing 10% D_2_O. ^1^H,^15^N heteronuclear single quantum coherence (HSQC) spectra of the BRD4 BD1 and BD2 were collected while SRX3305 was titrated in the NMR samples.

### Cell viability assay

JeKo-1 (parental/WT BTK or BTK C481S mutant), Mino (parental/WT or BTK C481S mutant), and Granta cells were plated in 96 well plates at a density of 2x10^4^ in 100 μL media and incubated overnight. Cells were treated with DMSO (0.1% final) or increasing concentrations of the indicated inhibitors for 48 h. 100 μL of Alamar blue (ThermoFisher Scientific) and Cell titer Glow assay reagent (Promega Cat # G9241) was added to each well, and the cells were incubated for 10 min at room temperature. Luminescence signal was measured in a VarioScan multimode reader. For Alamar Blue, fluorescence signals were read (emission at 590 nm after excitation at 560 nm). Averaged values were normalized as a percentage of DMSO (control) and analyzed by nonlinear regression then plotted for dose response using GraphPad Prism (GraphPad Software, Inc.). Experiments were performed in triplicate.

### Cytotoxicity assays using non-tumor cells

MTS [3-(4,5-dimethylthiazol-2-yl)-5-(3-carboxymethoxyphenyl)-2-(4-sulfophenyl)-2H-tetrazolium] assays were used to determine inhibitor-induced cytotoxicity. Briefly, healthy donor primary cells (~0.7e6/well) or HS-5 stromal cells (~25,000/well) were treated with vehicle (DMSO) or increasing inhibitor concentrations for up to 72 h in 96-well plates and then the CellTiter 96® AQueous assay (Promega, Madison, WI) was preformed according to manufacturer's instruction to determine cell proliferation. Absorbance signal from each well was acquired at 490 nm on a Tecan Infinite® M1000 Pro microplate reader (Männedorf, Switzerland).

### *Ex vivo* proliferation assay using primary E*μ*-Myc tumor samples

Spleens were harvested from terminally diseased E*μ*-Myc mice (Jackson Laboratory) housed at the Animal Research Facility (UNMC), adhering to institutional animal care guidelines. Murine splenocytes (~0.7e^6^/well) were stimulated with 1X PMA/ionomycin (BioLegend) and treated with vehicle (DMSO), ibrutinib, idelalisib, JQ-1, or SRX3305 for 48 h in 96-well plates. The CellTiter 96® AQ_ueous_ assay (Promega, Madison, WI) was then preformed according to manufacturer's instructions to determine cell proliferation. Absorbance signal from each well was acquired at 490 nm on a Tecan Infinite® M1000 Pro microplate reader (Männedorf, Switzerland).

### Western blot

JeKo-1, Mino (parental/WT or BTK C481S mutant) and Granta cells were plated in 10 cm tissue culture dishes at a cell density of 2 x 10^6^ and were incubated overnight. The cells were serum starved for 6 h and then treated in the presence of increasing concentration of SRX3305 or Ibrutinib for 1 h, followed by stimulation with 10 μg/mL of goat F(ab) 2 anti-human antibody (Southern Biotech, Birmingham, Cat # 2022-01) for 15 min at 37 °C. Whole cell lysates of the treated and untreated JeKo-1 and Mino cells were prepared using RIPA buffer supplemented with protease, phosphatase/protease inhibitor cocktails (Thermo Scientific) and 0.1% NP40. Protein concentration in cell lysates was determined using a bicinchoninic acid (BCA) assay kit (Thermo Fisher). Equal amounts of lysate were resolved on a 4-12% SDS-PAGE, transferred to nitrocellulose membranes, and probed with one or more of the following antibodies: anti-pBTK-Y223 (Cat # 5082S), anti-pAKT-S473 (Cat # 9271S), total anti-BTK (Cat # 8547S), total anti-AKT (Cat # 4691), and anti-β-actin (Cat # SC69879). Secondary antibodies were chosen according to the species of origin of the primary antibody. Protein bands were detected using the Pierce enhanced chemiluminescence (ECL) substrate (Thermo Fisher) and imaging system. Uncropped western blots images are shown in Supplementary Fig. 7.

### FITC Annexin V apoptosis assay

Jeko-1 and Mino cells were seeded at a density of 2x10^6^ cells and incubated overnight. Cells were treated with the specified concentrations of Ibrutinib, SRX3305, SRX3262 or controls DMSO and Staurosporine and incubated for 24 h. Cells were harvested and washed twice with cold PBS, and then labeled with Annexin V-FITC and PI (BD Pharmingen, Cat # 556419) or Annexin APC (Thermo Fisher, Cat # A35110) according to the manufacturer’s protocol (BD Pharmingen). The labeled cells were analyzed using BD Accuri C6 Flow cytometer. Experiments were performed in duplicate.

### RNA isolation and qRT-PCR

5x10^6^ Jeko-1 cells were seeded in 10 cm plate and incubated overnight. The cells were treated with 1 μM PLX51107 (BRD4 Inhibitor), 0.5 μM SRX3305 or DMSO for 24 hr. RNA was extracted post 24 hr by RNeasy mini kit (Qiagen). 1 μg of total RNA was converted to cDNA using iScript cDNA synthesis kit (BIO-RAD). cDNA amplification was performed with 1×SYBR green supermix on a QuantStudio™ 3 Real-Time PCR System. cDNAs were amplified using specific *cMYC* and *HEXIM1* primers, and the data were normalized to GAPDH.

### Cell cycle analysis

JeKo-1 and Mino cells were treated with the specified concentrations of SRX3305, SRX3262, or Ibrutinib for 24 h. Cells were harvested, washed once with PBS, and then fixed with 70% ethanol and stored at -20 ^°^C overnight. The fixed cells were collected by centrifugation, washed twice with PBS, resuspended with assay buffer (PI-100 μL/mL, Triton-X-50 μL/mL and RNase A-100 μg/mL), and incubated in the dark for 30 min at RT. The samples were analyzed using BD Accuri C6 Flow cytometer. Experiments were performed in duplicate.

### BCR Pathway stimulation

JeKo-1, Mino (parental/WT or BTK C481S mutant) and Granta cells (1x 10^6^) were serum starved for 6 h and then incubated with or without SRX3305, SRX3262, Ibrutinib or controls for 1 h. Treated cells were then resuspended in 1 mL of RPMI-1640, supplemented with 20% FBS and stimulated with 10 μg/mL of goat F(ab’)_2_ anti-human IgM antibody at 37 °C for 10 min. Stimulated cells were harvested and lysed with RIPA buffer followed by western blot analysis for pBTK and pAKT.

### Inhibitor washout assay

Cells were incubated with SRX3305, SRX3262, Ibrutinib or controls for 1 h, washed three times with PBS, and incubated in RPMI-1640 supplemented with 20% FBS and penicillin/streptomycin for 24 h. The BCR pathways in the washed cells were stimulated with 10 μg/mL of goat F(ab’)_2_ anti-human IgM antibody, and the cells were harvested and lysed. The resulting lysates were analyzed by western blot.

### Statistical analysis

All data were quantified and plotted using GraphPad Prism 8.0 and are presented as the means ± SEM of two or more experiments unless indicated otherwise. In cytotoxicity assays utilizing non-tumor cells and primary tumor differences in cell viability between conditions of interest were assessed using ANOVA models followed by Dunnett’s post-hoc test. Log-transformations of the data were applied when necessary before modeling to stabilize variances. *P* values < 0.05 were considered statistically significant.

## Supplementary Information

**Additional file 1: Supplementary Figure 1.** Anti-proliferative activity of Granta cells treated with increasing concentrations of SRX3305 or Ibrutinib. **Supplementary Figure 2.** SRX3305 inhibits BTK/PI3K signaling. **Supplementary Figure 3.** qRT-PCR analysis of *cMYC* and *HEXIM1* expression levels in JeKo-1 cells treated with DMSO, 1 μM PLX51107 (BRD4 inhibitor) or 0.5 μM SRX3305. **Supplementary Figure 4.** SRX3305 induces apoptosis in JeKo-1 cells. **Supplementary Figure 5.** SRX3305 induces apoptosis in Mino cells. **Supplementary Figure 6**. Cell cycle arrest in Jeko-1 and Mino cells. **Supplementary Figure 7.** Uncropped western blots.

## Data Availability

The structures of the described compounds used in this study are proprietary information of SignalRx Pharmaceuticals, Inc. (*Patent number WO2020023340A1*, 2020), and are not publicly available. All other data are available from the authors upon reasonable request.

## Abbreviations

MCL: Mantle cell lymphoma
BTK: Bruton’s tyrosine kinase
BCR: B-cell receptor
PI3K: Phosphatidylinositol-3 kinase
R/R: Relapsed/refractory
