# Supplementary material for: Combinatorial inhibition of BTK, PI3K-AKT and BRD4-MYC as a strategy for treatment of mantle cell lymphoma
Source: Mol Biomed. 2022 Jan 15;3:2. doi: 10.1186/s43556-021-00066-9 (PMC8760370; doi:10.1186/s43556-021-00066-9)
Supplement: Supplementary file 1 — Additional file 1: Supplementary Figure 1. Anti-proliferative activity of Granta cells treated with increasing concentrations of SRX3305 or Ibrutinib. Supplementary Figure 2. SRX3305 inhibits BTK/PI3K signaling. Supplementary Figure 3. qRT-PCR analysis of cMYC and HEXIM1 expression levels in JeKo-1 cells treated with DMSO, 1 μM PLX51107 (BRD4 inhibitor) or 0.5 μM SRX3305. Supplementary Figure 4. SRX3305 induces apoptosis in JeKo-1 cells. Supplementary Figure 5. SRX3305 induces apoptosis in Mino cells. Supplementary Figure 6. Cell cycle arrest in Jeko-1 and Mino cells. Supplementary Figure 7. Uncropped western blots. [file 43556_2021_66_MOESM1_ESM.docx]

**Supplementary Information**

**Combinatorial inhibition of BTK, PI3K-AKT and BRD4-MYC as a strategy for treatment of mantle cell lymphoma**

Kendra R. Vann^1,#^, Dhananjaya Pal^2,3,#^, Audrey L. Smith^4^, Namood-e Sahar^3^, Maddeboina Krishnaiah^2^, Dalia El-Gamal^4^ and Tatiana G. Kutateladze^1,*^

^1^Department of Pharmacology, University of Colorado School of Medicine, Aurora, CO, USA

^2^Molecular Targeted Therapeutics Laboratory, Levine Cancer Institute, Charlotte, NC, USA

^3^Division of Hematology and Oncology, Department of Pediatrics, Moores Cancer Center, University of California San Diego, La Jolla, CA, USA

^4^Eppley Institute for Research in Cancer and Allied Diseases, Fred & Pamela Buffett Cancer Center, University of Nebraska Medical Center, Omaha, NE, USA

^#^Equal contribution

^*^Correspondence to: Tatiana G. Kutateladze, [tatiana.kutateladze@cuanschutz.edu](mailto:tatiana.kutateladze@cuanschutz.edu)

**
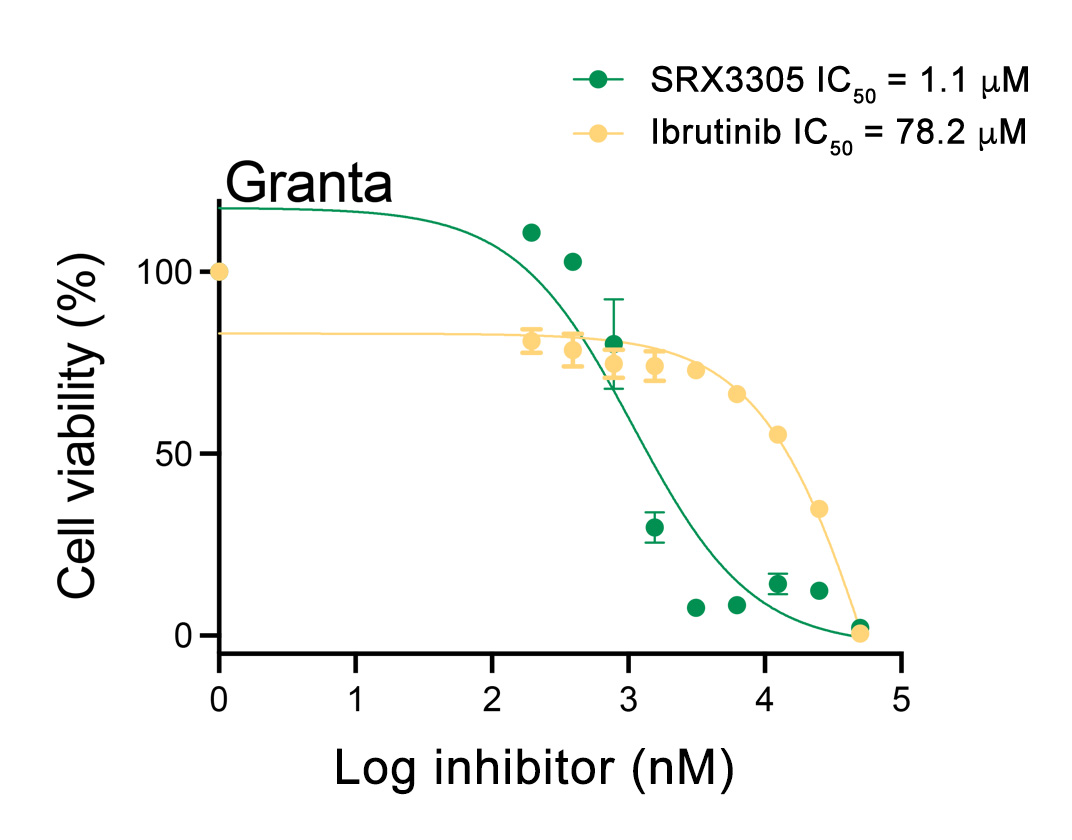
**

**Supplementary Figure 1.** Anti-proliferative activity of Granta cells treated with increasing concentrations of SRX3305 or Ibrutinib. The Granta cell line is a known Ibrutinib-resistant cell line that does not contain the C481S mutation in BTK. Error bars represent ± SEM derived from mean of triplicate experiments.

**
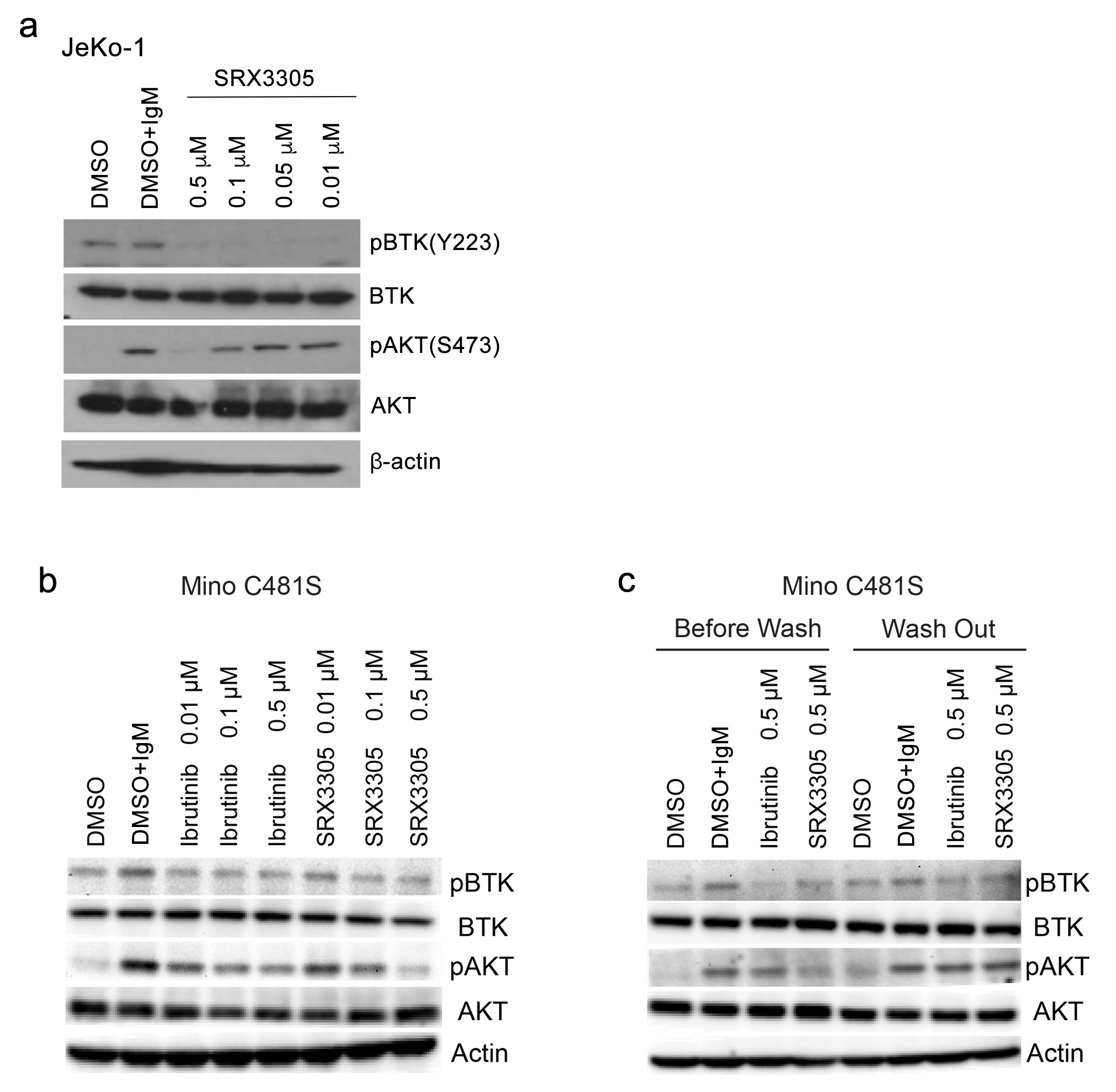
**

**Supplementary Figure 2.** SRX3305 inhibits BTK/PI3K signaling. (a) Western blot analysis of lysates from IgM-stimulated JeKo-1 cells treated with increasing concentrations of SRX3305 for 1 h. BTK and PI3K signaling was assessed by the levels of BTK, phosphorylated at Y223 BTK [pBTK(Y223)], AKT, and phosphorylated at S473 AKT [pAKT(S473)]. (b, c) Western blots analysis of lysates from IgM-stimulated Mino BTK C481S cells without and with washing out indicated inhibitors 24 h post treatment. Note that endogenous BTK is present in these cells.

**
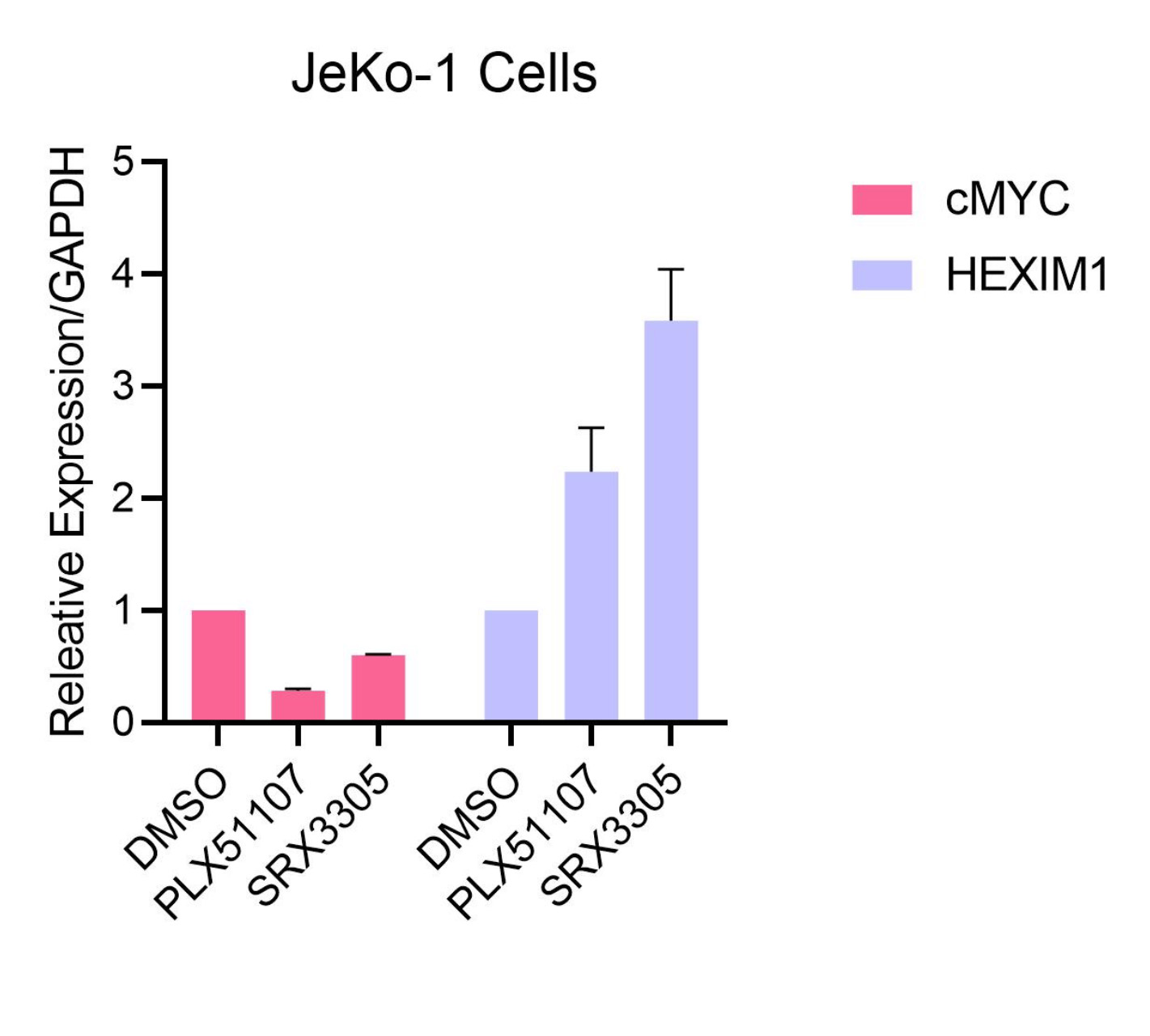
**

**Supplementary Figure 3.** qRT-PCR analysis of *cMYC* and *HEXIM1* expression levels in JeKo-1 cells treated with DMSO, 1 µM PLX51107 (BRD4 inhibitor) or 0.5 µM SRX3305. Error bars represent ± SD from the mean value of triplicates from two independent experiment.

**
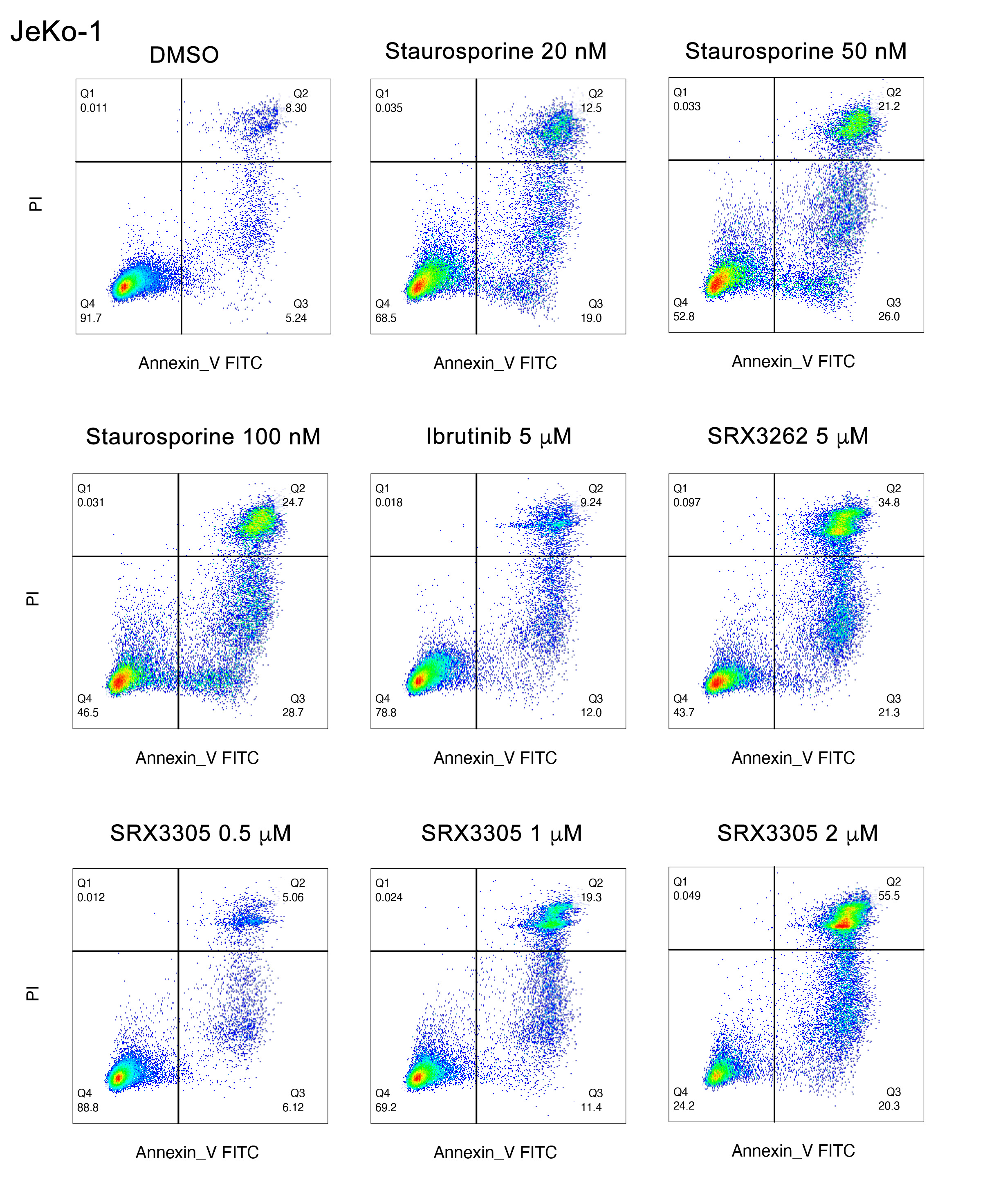
**

**Supplementary Figure 4.** SRX3305 induces apoptosis in JeKo-1 cells. Replicates of flow cytometry apoptosis analysis in the JeKo-1 MCL cell line treated with DMSO, Staurosporine, Ibrutinib, SRX3262 and SRX3305 for 24 h and assessed using Annexin V/ Propidium Iodide staining.

**
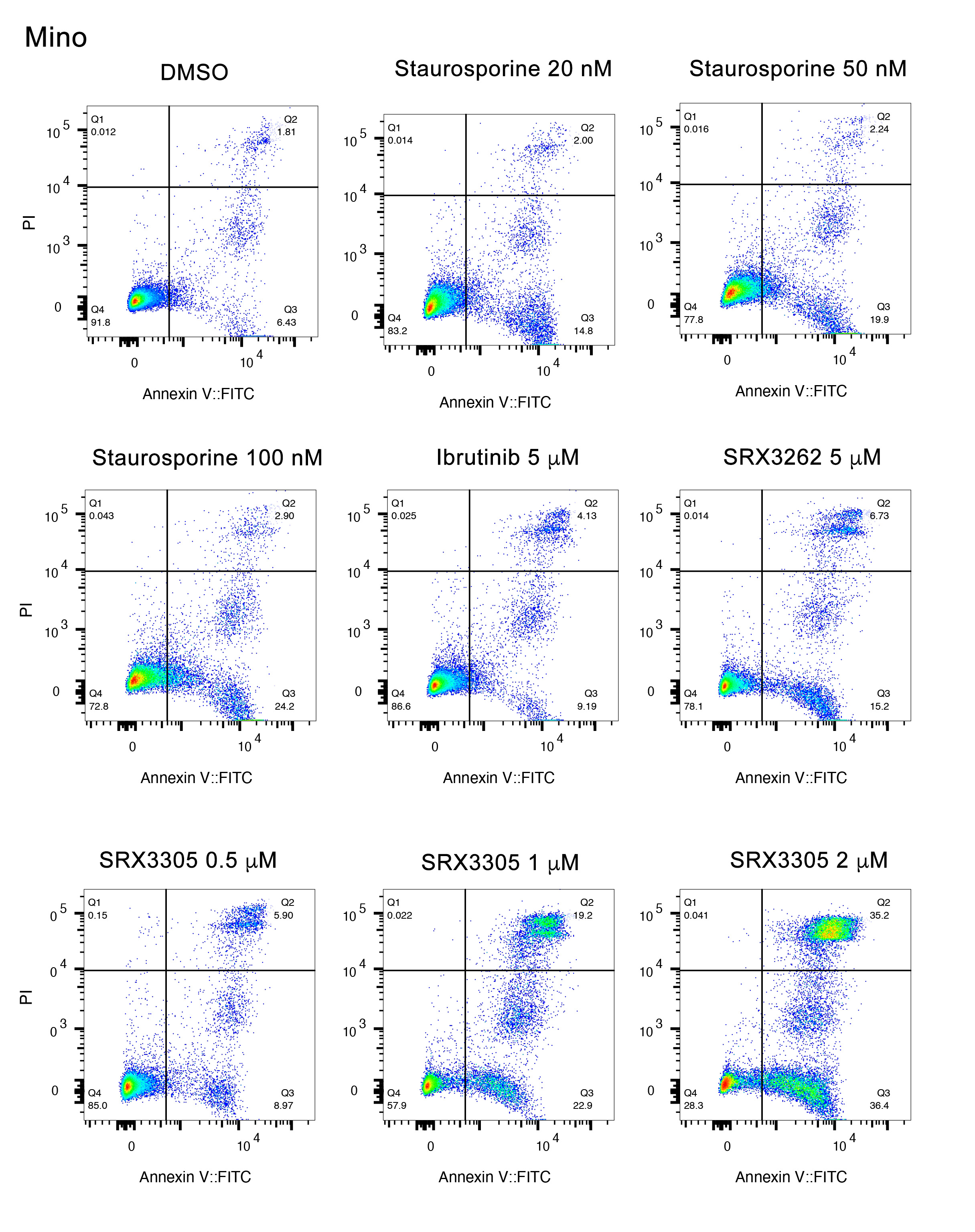
**

**Supplementary Figure 5.** SRX3305 induces apoptosis in Mino cells. Replicates of flow cytometry apoptosis analysis in the Mino MCL cell line treated with DMSO, Staurosporine, Ibrutinib, SRX3262 and SRX3305 for 24 h and assessed using Annexin V/ Propidium Iodide staining.


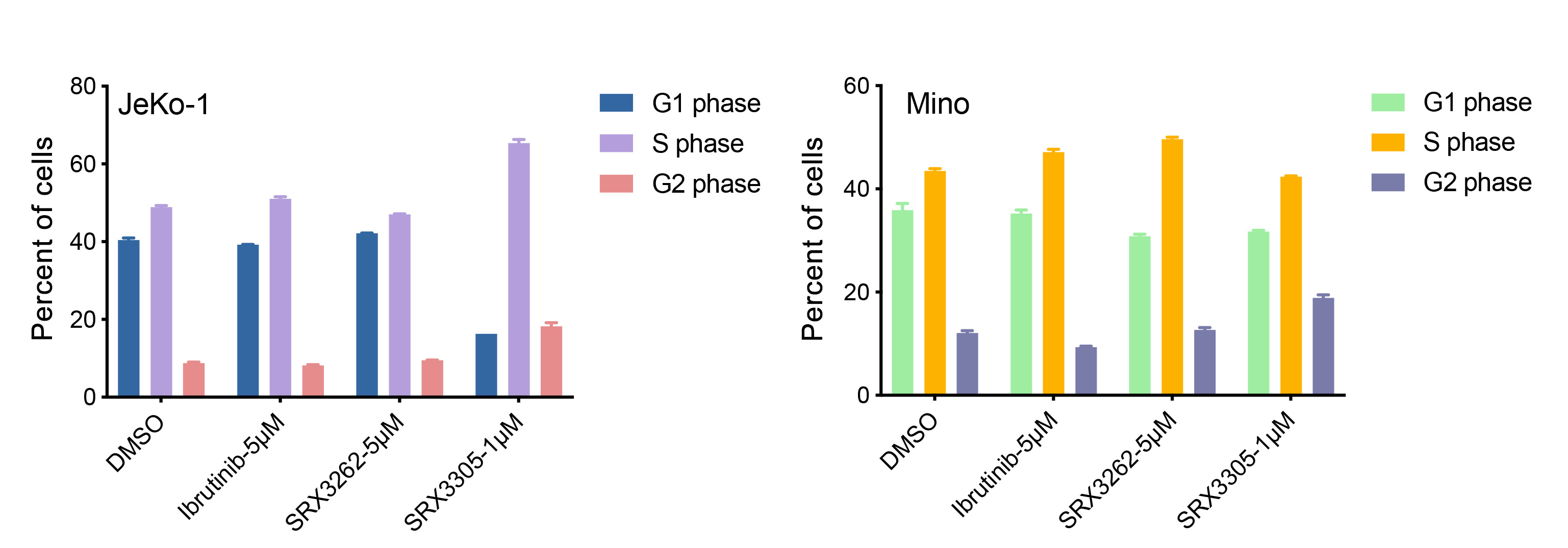


**Supplementary Figure 6**. Cell cycle arrest in Jeko-1 and Mino cells. Flow cytometry analysis of cell cycle arrest 24 h after Ibrutinib (5 µM), SRX3262 (5 µM) or SRX3305 (1 µM) treatment of JeKo-1 and Mino cells. The error bars represent SEM of the mean value from two independent experiments.

**
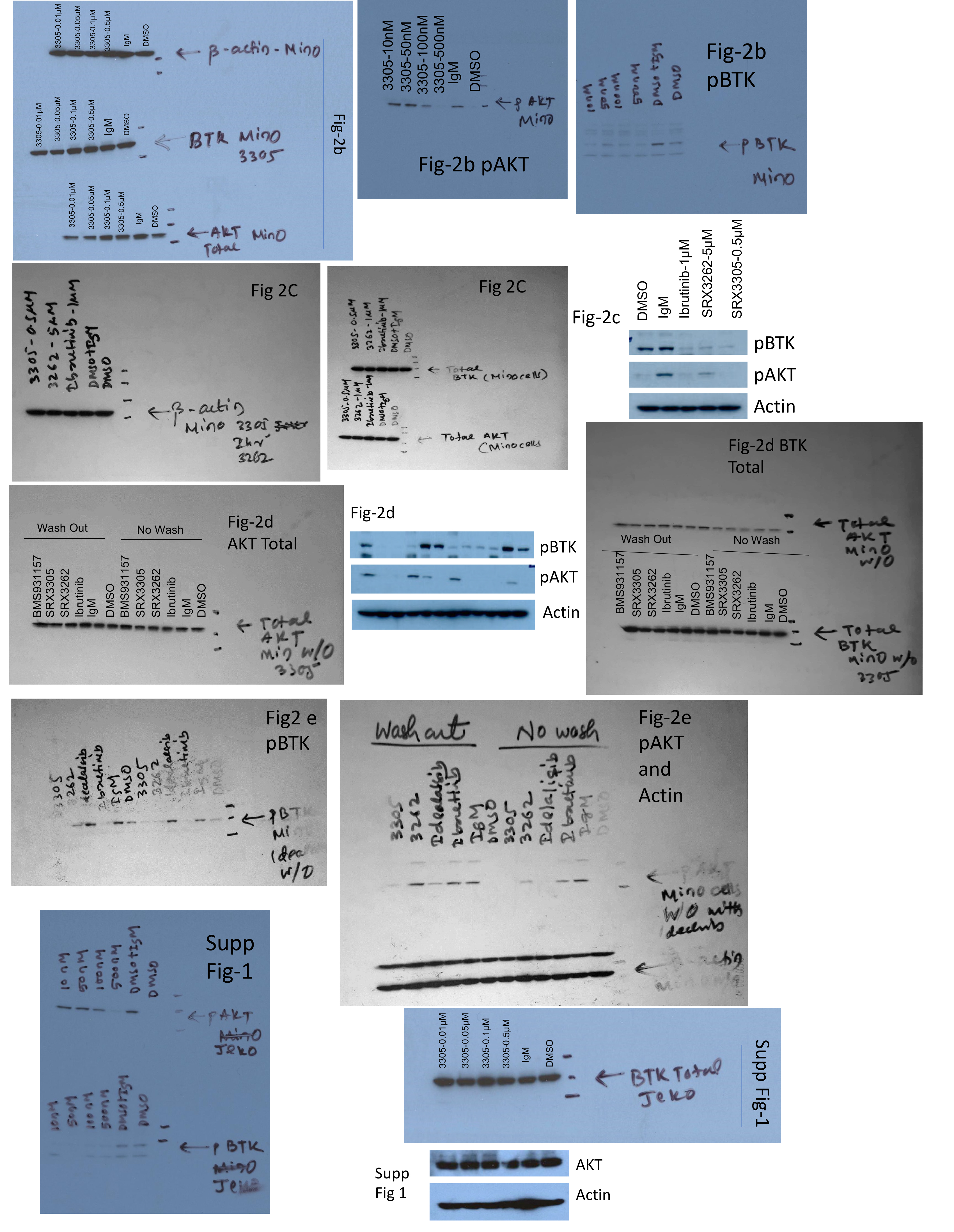
**

**Supplementary Figure 7.** Uncropped western blots.
